# ABA Is Involved in Regulation of Cold Stress Response in Bermudagrass

**DOI:** 10.3389/fpls.2017.01613

**Published:** 2017-10-13

**Authors:** Xuebing Huang, Haiyan Shi, Zhengrong Hu, Ao Liu, Erick Amombo, Liang Chen, Jinmin Fu

**Affiliations:** ^1^Key Laboratory of Plant Germplasm Enhancement and Specialty Agriculture, Wuhan Botanical Garden, Chinese Academy of Sciences, Wuhan, China; ^2^University of Chinese Academy of Sciences, Beijing, China; ^3^College of Horticulture, Agricultural University of Hebei, Baoding, China

**Keywords:** abscisic acid, Bermudagrass, cold stress, photosystem II, δ13C

## Abstract

As a representative warm-season grass, Bermudagrass [*Cynodon dactylon* (L). *Pers*.] is widely used in turf systems. However, low temperature remarkably limits its growth and distribution. ABA is a crucial phytohormone that has been reported to regulate much important physiological and biochemical processes in plants under abiotic stress. Therefore, the objective of this study was to figure out the effects of ABA on the cold-sensitive (S) and cold-resistant (R) Bermudagrass genotypes response to cold stress. In this study, the plants were treated with 100 μM ABA solution and exposed to 4°C temperature. After 7 days of cold treatment, the electrolyte leakage (EL), malonaldehyde (MDA) and H_2_O_2_ content were significantly increased in both genotypes compared with control condition, and these values were higher in R genotype than those of S genotype, respectively. By contrast, exogenous ABA application decreased the electrolyte leakage (EL), MDA and H_2_O_2_ content in both genotypes compared with those plants without ABA treatment under cold treatment condition. In addition, exogenous ABA application increased the levels of chlorophyll *a* fluorescence transient curve for both genotypes, and it was higher in R genotype than that of S genotype. Analysis of photosynthetic fluorescence parameters revealed that ABA treatment improved the performance of photosystem II under cold condition, particularly for the R genotype. Moreover, cold stress significantly increased δ13C values for both genotypes, while it was alleviated by exogenous ABA. Additionally, exogenous ABA application altered the expression of ABA- or cold related genes, including *ABF1*, *CBF1*, and *LEA*. In summary, exogenous ABA application enhanced cold resistance of both genotypes by maintaining cell membrane stability, improving the process of photosystem II, increasing carbon isotopic fractionation under cold stress, and more prominently in R genotype compared with S genotype.

## Introduction

Bermudagrass [*Cynodondactylon* (L) *Pers*.] is widely used in golf courses, sports fields and lawns globally (Fan et al., 2014). As a representative warm-season grass, its optimal growth temperature ranges from 26°C to 35°C (Fan et al., 2014). Cold stress is considered to be a key environmental factor that limits its growth and distribution (Fan et al., 2014).

When two Bermudagrass [*Cynodon dactylon* (L.) Pers. var. *dactylon*] cultivars, Riviera (cold tolerant) and Princess-77 (cold sensitive) were exposed to cold acclimation at 8/4°C (day/night), relative EL values were remarkably increased, resulting in the damage to cell membrane (Zhang et al., 2006). Previous results showed that H_2_O_2_ content increased in plants tissues after low temperature treatment (Fadzillah et al., 1996; O’Kane et al., 1996). To protect themselves from cold-induced damage, plants have evolved multiple mechanisms to enhance their cold tolerance, which include alterations of membrane fluidity, metabolism homeostasis, enzyme activity and the C-repeat-Binding Factor/DRE-Binding Factor (CBF/DREB) pathway (Thomashow, 1999; Zhu et al., 2004; Chinnusamy et al., 2010; Knight and Knight, 2012).

Photosynthesis plays a vital role in photochemical and biochemical process, which transforms solar energy into chemical energy of biomass production, but it is highly sensitive to low temperature. Previous studies suggested that when rice were exposed to cold conditions of 5, 10, 15, 20, and 25°C for 16 h, there was a steady decline in photosynthesis of cold-sensitive rice cultivars ‘Milyyang23,’ compared to that of cold-resistant ‘Stejaree45’(Jeong et al., 2002). Krause (1994) reported that under cold stress, the efficiency of photosynthesis electron transport decreased notably in plant, which resulted in excessive energy generation. Generally, when plants are subjected to photo inhibitory conditions, reactive oxygen species (ROS) are formed, which leads to severe injury of PS II components (Osmond, 1994). The extent of inactivation relies on the balance between inactivation and re-synthesize of PS II components during the low temperature stress (Allakhverdiev et al., 2008; Chen et al., 2013).

As we all know that plants will change the way of carbon metabolism if photosynthesis is affected. There are two naturally stable isotopes of carbon, ^12^C and ^13^C. Majority of the carbon is ^12^C (98.9%), with 1.1% being ^13^C (O’Leary, 1988). The distribution of isotopes among different compounds can reveal information about the physical, chemical and metabolic processes involved in carbon transformations (Farquhar et al., 1989). Recently, stable carbon isotope analysis is a rapidly developing technique which may reveal many processes of carbon dynamics in plants. Sustaining this technological trend will enable researchers to discover how plants respond to abiotic and biotic stress (Dawson et al., 2002). Previous studies reported that conditions triggering stomatal closure, such as salinity, water stress and decrease the CO_2_ supply to carboxylation sites increased the δ13C of plants (Rivelli et al., 2002; Araus et al., 2003; Yousfi et al., 2010), but the evaluation of whether variation in δ13C is the result of changes in intrinsic photosynthetic capacity or stomatal conductance remains unknown (Scheidegger et al., 2000; Farquhar et al., 2007).

ABA is a vital phytohormone that regulates many essential physiological and biochemical processes, and it has a key role in stress resistance during plant growth and development (Verslues and Zhu, 2005; Fujii et al., 2009; Kim et al., 2016). Previous study suggested that cold stress is accompanied by increased levels of endogenous ABA in many plants (Mantyla et al., 1995), and exogenous ABA treatment could enhance plant cold resistance (Kumar et al., 2008; Kim et al., 2016). Under low temperature condition, plants activate downstream gene expression through both ABA-dependent and ABA-independent pathways. In Arabidopsis, the expression levels of ABA responsive transcription factor, *ABF1* and *ABF4*, were induced under cold condition (Choi et al., 2000). Furthermore, exogenous application ABA also increased the content of soluble sugar and proline, improved water retention (Deng et al., 2005; Huang et al., 2015), reduced membrane lipid peroxidation, alleviated cell membrane injury effectively (Zhou and Guo, 2005; Huang et al., 2015), and enhanced photosynthesis (He et al., 2008).

Recently, profound progress has been made in investigating ABA participation in abiotic stress response, while studies about the effects of exogenous ABA treatment under cold conditions in Bermudagrass species are still very limited. Here, we aim at investigating the possible cold adaptive role of ABA in the cold-sensitive and cold- resistance Bermudagrass genotypes, particularly focusing on cell membrane stability, photosynthesis and stable isotope signatures. This study provides some insights into the possible physiological and molecular mechanisms underlying Bermudagrass response to cold stress.

## Materials and Methods

### Plant Materials and Growth Conditions

Two genotypes of Bermudagrass were used in this study, they are cold-resistant ‘WBD128’ and cold-sensitive ‘WBGg-17,’ which were collected from Xiaojiang city, Zhengjiang province, China (N 27°34.258, E 106°27.383). The Bermudagrass stolons were planted in the plastic pots (7.5 cm in diameter and 9.0 cm deep) that were filled with solid growth substances (brown coal soil). Several drainage holes were drilled at the bottom of the pots to avoid excessive water accumulation and to enhance soil aeration. For Bermudagrass establishment, the pots were maintained in the greenhouse with the temperature regime of 30/25°C (day/night) for about 2 months. During the establishing period, the plants were watered three times each week and fertilized weekly with full-strength Hoagland’s solution.

### Treatments

After establishment, the grasses were transferred into the growth chamber (LSC-339CF, Xingxing group Co., Zhejiang, China) with different conditions. The condition of control chamber was 12 h photoperiod and 28/24°C (day/night) and 70% relative humidity. The cold-treated chamber conditions were similar to the control chamber, except for 4°C (day/night) temperature. Two Bermudagrass genotypes were grouped into three regimes, as follows: (i) the cold-sensitive/resistance genotype were subjected to 28°C/24°C (day/night) (CK); (ii) the cold-sensitive/resistance genotype were subjected to 4°C (LT); (iii) the cold-sensitive/resistance genotype treated with 100 μM ABA solution at 4°C (LTA). All treatments were kept in the growth chamber for 7 days, and during the treatment period, Bermudagrass was watered every 3 days with ABA solution.

### Measurement of Electrolyte Leakage

For the determination of EL, after 7 days treatment, about 0.1 g of fully extended leaf samples were collected from the Bermudagrass and washed three-times with deionized water, and then the leaves were cut to about 0.5 cm long segments and then were transferred into a 50 mL plastic centrifuge tube that was filled with 15 mL of deionized water. The centrifuge tube with fragments were shaken for 24 h at room temperature and the primary conductively (EL_1_) was measured with a conductivity meter (JENCO-3173, Jenco Instruments, Inc., San Diego, CA, United States). Subsequently, in order to disrupt the tissues and release all the electrolytes into to the solution completely, the leaf tissues in the tube were heated at 121°C for 30 min. After the samples had cooled to room temperature, the second conductively (EL_2_) was measured. The relative EL was calculated by using the equation:

Relative⁢ EL(%)=EL1EL2×100

### Crude Enzyme Extraction

To extract crude enzyme, about 0.2 g of fully extended leaves were collected and inserted into liquid nitrogen immediately. Subsequently, the leaf samples were ground into powder with liquid nitrogen in the mortar (pre-cooled at 4°C), 4 mL of 150 mM pre-cooled sodium phosphate buffer (Ph7.0) was added into the powder. Then the homogenate was transferred into 15 mL centrifuge tube, and centrifuged with 12000 rpm for 20 min at 4°C, the supernatant fluid was collected for physiological assays, including the H_2_O_2_, and malonaldehyde (MDA) contents.

### Determination of MDA Content

Malonaldehyde content was measured by the thiobarbituric acid (TBA) method (Fan et al., 2014) with slight modifications. About 1ml of crude enzyme was mixed with 2 mL MDA reaction solutions that included 20% (v/v) trichloroacetic acid (TCA) and 0.5% (v/v) TBA. The mixed solution was heated for 30 min at 95°C in the water bath, then fast-cooled to room temperature and centrifuged at 20°C with 12000 rpm for 10 min. The MDA content was determined by measuring the absorbance at 532 and 600 nm using a spectrophotometer (UV-2600, UNICO Instruments Co. Ltd., Shanghai, China), and was calculated according to the formula:

$$MDA(nmol\,\;g^{-1}\,\;FW)=\frac{\left[\left(OD532-OD600\right)\right]}{\left(l\times \in \;FW\right)}\times 12$$

Where L was the volume of the crude enzyme; l refers to the thickness of the cuvettes; ε indicates the extinction coefficient of 155 mM^-1^cm^-1^.

### Assay of H_2_O_2_ Content

About 100 μL of crude enzyme solution was used to measure H_2_O_2_ content, which was measured by monitoring the absorbance at 405 nm according to the method of NJBI (Nanjing Jiancheng Bioengineering Institute) determination kit with Multiskan Spectrum Microplate Spectrophotometer (M200 PRO, Austria).

### Chlorophyll *a* (Chl*a*) Fluorescence Transient

All chlorophyll *a* (Chl*a*) fluorescence transient curves were measured by pulse-amplitude modulation (PAM) fluorimeter (PAM2500, Heinz Walz GmbH) with high time resolution (10 μs). After a dark-adaptation for 30 min, the OJIP transients were triggered by a red light of 3000 μmol photons m^-2^s^-1^, which was guarantee closure of all photosystem II (PS II) reaction centers (RC) to obtain a true maximal fluorescence excitation intensity (Chen et al., 2014). Subsequently, the Chl*a* fluorescence transient was triggered by the strong light, and then was measured and digitized between 10 μs and 320 ms. The OJIP transients were analyzed using the JIP-test method described by Yusuf et al. (2010).

### Assay of Stable Isotope of Carbon

About 0.1 g of fresh leaves was collected from the plants, oven dried and ground. The stable carbon (^13^C:^12^C) isotope ratios in the leaves were determined using a Stable Isotope Mass Spectrometer (delta v advantage, Germany) in continuous flow mode. About 0.2–0.3 mg samples and reference materials were weighted into tin capsules, sealed and put into an automatic sampler. Values were calculated according to this formula (Coplen, 2008):

$$\delta^{13}C=\frac{{\left(13C/12C\right)}_{sample}-{\left(13C/12C\right)}_{s\,\tan dard}}{{\left(13C/12C\right)}_{s\,\tan dard}}\times 1000$$

Where ‘sample’ refers to Bermudagrass and ‘standard’ to Pee Dee Belemnite (PDB) calcium carbonate.

### Quantitative RT-PCR Analysis

For gene expression determination, about 0.1 g of fresh leaves was collected from the Bermudagrass. Total RNA was extracted using Trizol-reagent (Invitrogen, Carlsbad, CA, United States).

The first strand was synthesized from 2.5 μg total RNA with oligo (dT)_12-18_ primer. The gene-specific primers used in this study were listed in **Table 1**, actin was used as control. The quantitative real time-PCR was performed on the StepOnePlus Real-Time PCR Systems (Applied Biosystems, United States). Three technical replications for each sample were performed.

**Table 1:** Primer sequences used for the expression of genes.

Gene	Primer sequences
ABF1-F	AATGGATTGGTGACGGGAG
ABF1-R	CATTGAAAACGTATGGCACTGG
CBF1-F	ACCAAGTTCCGCGAGACGC
CBF1-R	CGAGTCGGCGAAGTTGAGGCA
LEA-F	TCATCCCCAGCGTGTTCATCA
LEA-R	GAGGCCGCCAAACAGAAGACA
ACTIN2-F	TCTGAAGGGTAAGTAGAGTAG
ACTIN2-R	ACTCAGCACATTCCAGCAGAT


### Statistical Analysis

Each treatment was repeated three times, and values were given as mean ± SD. Statistical analysis was performed by using One-way ANOVA, and Duncan’s multiple range test was used to separate means at a significant level of *P* < 0.05, using the statistical package SPSS20.0 and Excel 2010 for windows.

## Results

### Growth Phenotype

**FIGURE 1:**
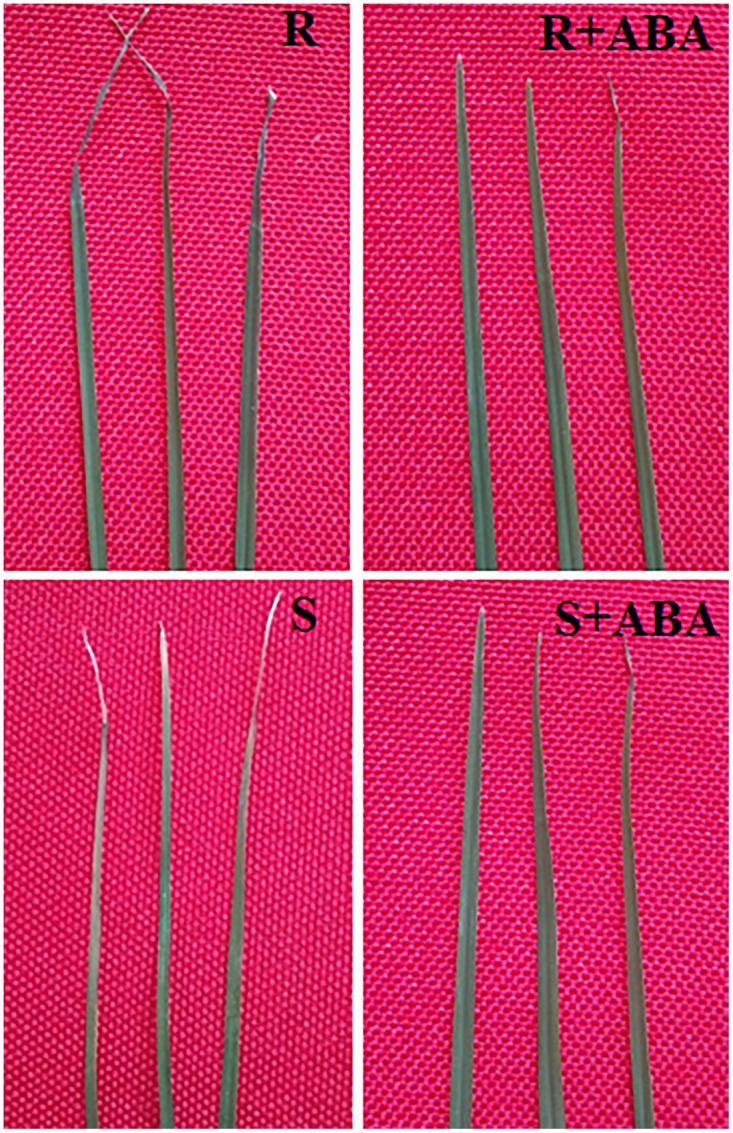
Effects of ABA application on growth phenotype of Bermudagrass after 7 days of cold stress (4°C). R and S represented cold-resistance and cold-sensitive Bermudagrass genotype, R + ABA and S + ABA represents the treatment with 100 μM ABA solution at 4°C (day/night), respectively.

The results showed that after 7 days cold stress, without ABA treatment showed yellowed and rolled of leaf, and the S genotype was more serious than that of R genotype while ABA treatment showed alleviated phenotype (**Figure 1**). These findings indicated that ABA treatment improved Bermudagrass tolerance to cold stress, especially in cold- resistant Bermudagrass.

### Cell Membrane Stability and Lipid Peroxidation

**FIGURE 2:**
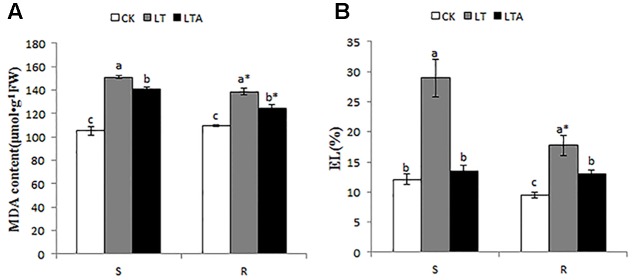
Alteration of cell membrane stability and lipid peroxidation in the leaves of Bermudagrass after 7 days of different treatment. **(A)** Malonaldehyde content; **(B)** electrolyte leakage. The CK represents control treatment at the optimum temperature (28/24°C, day/night). LT refers to cold treatment at a temperature of 4°C (day/night). LTA represents the treatment with 100 μM ABA solution at a temperature of 4°C (day/night). S and R represent cold-sensitive and cold-resistance Bermudagrass genotype, respectively. Mean and SD were calculated from three repeats of each treatment. Columns marked with different letters indicate significant statistical differences among different regimes at different Bermudagrass genotype based on Ducan’s multiple range tests (*P* < 0.05). Columns marked with star represent statistical significance between R and S for a given regime based on Independent-samples *t*-test (*P* < 0.05).

To investigate whether the exogenous ABA played a positive role in maintaining cell membrane stability of Bermudagrass species under cold stress, MDA content and EL levels were determined. It was evident that the levels of EL and MDA content were higher in both genotypes under cold treatment compared with normal temperature, and the level in the S genotype was higher than that of R genotype. After ABA treatment, EL levels were 15.39% (S) and 4.68% (R) lower than those of plants without ABA treatment. Similarly, the MDA content in Bermudagrass treated with ABA were 6.5% (S) and 10.1% (R) lower than those without ABA treatment, respectively, and the levels in the R genotype were 11.9% lower than that of S genotype after ABA treatment (**Figure 2**). These results indicated that there was more cell membrane damage in the S genotype than the R genotype, and exogenous application of ABA significantly improved cell membrane stability, especially in cold- resistant Bermudagrass.

### H_2_O_2_ Content

**FIGURE 3:**
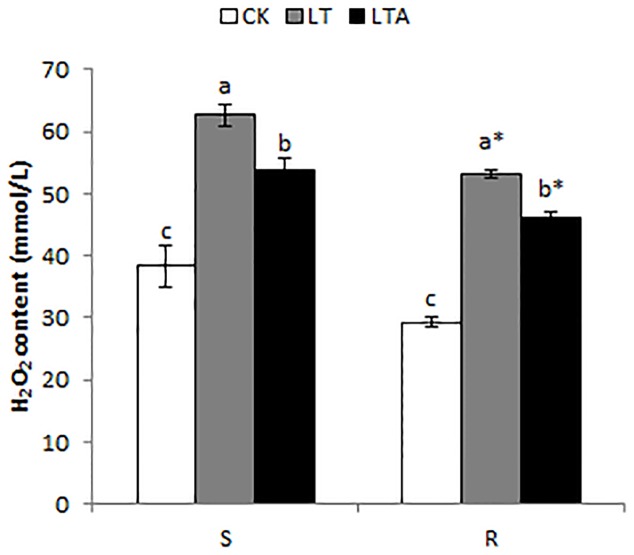
Alterations of H_2_O_2_ content in the leaves of Bermudagrass after 7 days of different treatment. There were three repeats of each treatment. The CK represents control treatment at the optimum temperature (28/24°C, day/night). LT refers to cold treatment at a temperature of 4°C (day/night). LTA represents the treatment that treated with 100 μM ABA solution at a temperature of 4°C (day/night). S and R represented cold-sensitive and cold-resistance Bermudagrass genotype, respectively. Mean and SD were calculated from three repeats of each treatment. Columns marked with different letters indicate significant differences among different regimes at different genotype based on Ducan’s multiple range tests (*P* < 0.05). Columns marked with star represent statistical significance between R and S for a given regime based on Independent-samples t test (*P* < 0.05).

In the present study, the extent of oxidative damage to cell membrane triggered by cold stress was investigated through H_2_O_2_ content measurement. Compared with normal temperature, cold stress significantly increased H_2_O_2_ content, and the level in the S genotype was 15.2% higher than that of the R genotype. Exogenous application of ABA dramatically decreased the level of H_2_O_2_ under cold stress in both genotypes, and the H_2_O_2_ content were 14.0% (S) and 13.1% (R) lower than that without ABA treatment respectively. In addition, the concentration in the R genotype was 14.3% lower than S genotype (**Figure 3**). These results showed that exogenous application of ABA could decrease oxidative damage of both genotypes caused by cold.

### OJIP Transient Curves and JIP-Test

**FIGURE 4:**
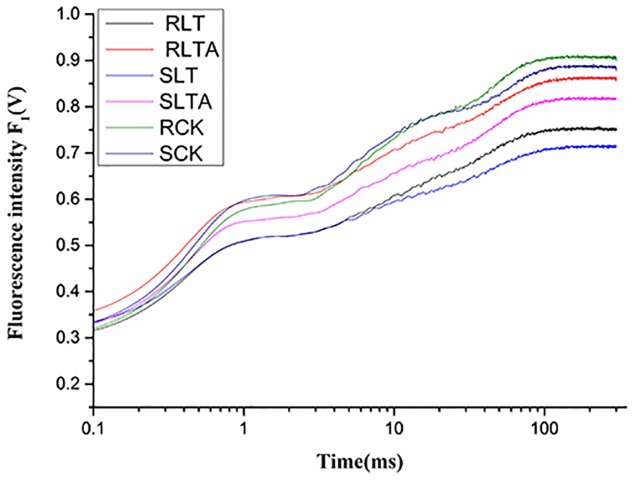
Alteration of chlorophyll fluorescence transients in the leaves of Bermudagrass after 7 days of different treatment. The CK represents control treatment at the optimum temperature (28/24°C, day/night). LT refers to cold treatment at a temperature of 4°C (day/night). LTA represents the treatment that treated with 100 μM ABA solution at a temperature of 4°C (day/night). S and R represented cold-sensitive and cold-resistance Bermudagrass genotype, respectively.

To investigate the effect of ABA on photosynthesis system, the OJIP fluorescence transient curves was measured (**Figure 4**). After 7 days of treatment, the OJIP curves of two Bermudagrass genotypes dropped dramatically under cold condition, and the S genotype showed lower levels than the R genotype. Moreover, the curves were higher in both genotypes with exogenous ABA than those without exogenous ABA application under cold condition, and the R genotype showed higher levels than the S genotype. These observations indicated that exogenous ABA treatment increased the OJIP fluorescence transient curves of both Bermudagrass, and more prominently in the R than the S genotype.

To further explore the structural and functional parameters quantifying the photosynthetic behavior of the Bermudagrass leaves under cold stress, the values of fluorescence parameters were analyzed by the JIP-test (**Table 2**). Performance index (PI), PI_total_ and PI_ABS_ are important indexes describing the overall activity of PS II. Cold stress dramatically reduced the Performance index values for both genotypes, which were relieved by ABA treatment. Similar to PI, ABA treatment increased the parameters of quantum yields and probabilities under cold stress, such as φP0 (Maximum quantum yield for primary photochemistry), φE0 (Quantum yield of the electron transport flux from Q_A_ to Q_B_) and 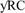 (Probability that a PS II Chl molecule functions as RC) values (**Table 2**). These results suggested that exogenous ABA played an active role of photosynthetic performance in Bermudagrass response to cold stress. ABS/RC (Absorbed photon flus per RC), TR0/RC [Trapped excitation flux (leading to QA reduction) per RC] and RE0/RC (Electron flux reducing end electron acceptors at the PS I acceptor side, per RC) values, also changed significantly after different treatment. Cold stress increased the values of ABS/RC and RE0/RC in both genotypes, which were alleviated by ABA treatment. However, low temperature enhanced the TR0/RC and ET0/RC values in cold-sensitive Bermudagrass, which reduced that of cold- resistant Bermudagrass (**Table 2**). These results indicated that cold stress might enhance the energy fluxes for absorption and trapping in both genotypes, which was eventually inhibited by exogenous ABA.

**Table 2:** Photosynthetic parameters deduced by the JIP-test analysis fluorescence transients.

Photosynthetic parameters	SM			RM			Definitions
			
	CK	LT	LTA	CK	LT	LTA	
**Specific energy fluxes (per active PS II reaction center)**							
ABS/RC	3.05b	3.64a	3.24b	2.90c	3.58a	3.28b	Absorbed photon flus per RC
TR0/RC	2.24c	2.67a	2.50b	2.19b	2.08c	2.39a	Trapped excitation flux(leading to QA reduction) per RC
ET0/RC	1.07b	1.35a	1.18b	1.08b	1.04c	1.22a	Electron transport flux(further than QA^-^) per RC
RE0/RC	0.42c	0.74a	0.61b	0.47b	0.52a	0.45c	Electron flux reducing end electron acceptors at the PS I acceptor side, per RC
**Quantum yields and efficiencies/probabilities**							
φP0	0.73a	0.67c	0.70b	0.75a	0.65c	0.68b	Maximum quantum yield for primary photochemistry, namely F_V_/F_M_
φE0	0.34a	0.31b	0.34a	0.37a	0.33b	0.34b	Quantum yield of the electron transport flux from Q_A_ to Q_B_
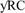	0.24a	0.21c	0.22b	0.25a	0.21c	0.22b	Probability that a PS II Chl molecule functions as RC
**Performance Indexes (PI, combination of parameters)**							
PI_ABS_	0.62a	0.38b	0.65a	0.99a	0.56c	0.64b	PI(potential) for energy conservation from exciton to the reduction of intersystem electron
PI_total_	0.54b	0.49c	0.73a	0.77a	0.45c	0.56b	PI(potential) for energy conservation from exciton to the reduction of PS I end acceptors


*The calculation of each parameter is based on the method described by Yusuf et al. (2010). Subscript “0” indicated that the parameter refers to the onset of illumination. Values are given as the average of 3 replicates, and different letters indicated statistical difference significance at *P* < 0.05 among the treatments by Ducan’s multiple range tests. The CK represents control treatment at the optimum temperature (28/24°C, day/night). LT refers to cold treatment at a temperature of 4°C (day/night). LTA represents the treatment that treated with 100 μM ABA solution at a temperature of 4°C (day/night). S and R represented cold-sensitive and cold-resistance Bermudagrass genotype, respectively.*

### δ13C Value

**FIGURE 5:**
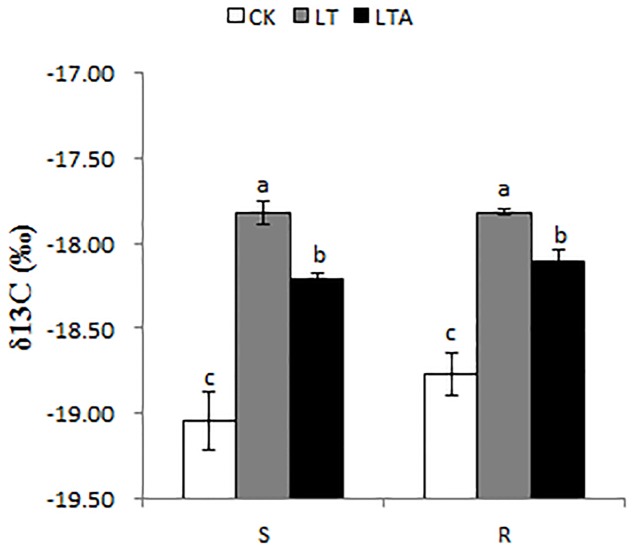
Effects on carbon isotope composition in the leaf Bermudagrass after 7 days of different treatment. There were four repeats of each treatment. The CK represents control treatment at the optimum temperature (28/24°C, day/night). LT refers to cold treatment at a temperature of 4°C (day/night). LTA represents the treatment with 100 μM ABA solution at a temperature of 4°C (day/night). S and R represented cold-sensitive and cold-resistance Bermudagrass genotype, respectively. Mean and SD were calculated from three repeats of each treatment. Columns marked with different letters indicate significant differences among different regimes at different Bermudagrass genotype based on Ducan’s multiple range tests (*P* < 0.05).

Carbon isotopic composition can be evaluated by the growth condition of plants, which further reflect the alterations of stoma and photosynthesis (Farquhar et al., 1982). In order to assess genotype responses of Bermudagrass under cold stress, δ13C value was measured. In this study, low temperature significantly increased the δ13C values in two genotypes compared to the normal temperature. However, the level of δ13C decreased in the absence of exogenous ABA under low temperature. Interesting, there were no difference in both genotypes (**Figure 5**).

### Quantitative RT-PCR Analysis of Cold/ABA-Related Genes

**FIGURE 6:**
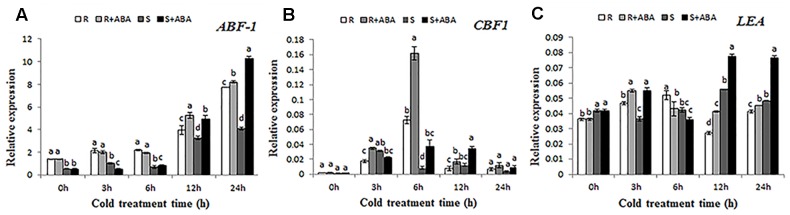
Effects of ABA treatment on expression level of stress inducible genes under cold stress (4°C). **(A)** Relative expression of *ABF1*; **(B)** Relative expression of *CBF1*; **(C)** Relative expression of *LEA*. Total RNA was isolated from Bermudagrass leaves treated at 4°C for 0, 3, 6, 12, and 24 h, respectively. Quantative real-time PCR was repeated for three times. R and S represented cold-resistance and cold-sensitive Bermudagrass genotype, RA and SA represents the treatment with 100 μM ABA solution at a temperature of 4°C (day/night) respectively. Mean and SD were calculated from three repeats of each treatment. Columns marked with different letters indicate significant differences among different regimes at different Bermudagrass genotype based on Ducan’s multiple range tests (*P* < 0.05).

To further explore how ABA modulates gene expression pattern of Bermudagrass in response to cold stress, expression of three stress inducible genes were analyzed. *ABF1* has been reported to play a critical role in cold stress tolerance. As shown in **Figure 6A**, after cold treatment, *ABF1* expression was significantly increased, and the cold-induced up-regulation was enhanced by ABA application at 12–24 h. In addition, the level in the R genotype was higher than that of S genotype at 3–12 h.

Cold stress activates the transcription of *CBF* and increase the expression of *COLD-REGULATED* (*COR*) genes (Liu et al., 1998), which enhance cold tolerance of plants. In the study, cold treatment significantly increased expression of *CBF1*, which reached a maximum at 6 h, and the level in the S genotype was lower than that of R genotype. After 6 h, the expression of *CBF1* began to down-regulate, but it was still higher than those of at 0 h (**Figure 6B**).

Late embryogenesis-abundant (LEA) protein has been reported to play an important role under cold conditions. As shown in **Figure 6C**, expression of *LEA* in R genotype was induced by both cold and ABA treatment at 3–6 h, decreased at 12 h, and then increased again at 24 h. On the contrary, for the S genotype, after cold treatment, the expression of *LEA* decreased at 3–6 h; however, ABA-treated Bermudagrass exhibited a higher level than those without ABA treatment at 3 h, and then increased again and maintained at a relatively higher level by both cold and ABA treatment at 12–24 h. Except for at 6 h, all the cold-induced up-regulation was enhanced by exogenous ABA application. Interestingly, the level of S genotype was higher than that of R genotype.

## Discussion

To survive extreme environmental conditions, botanical species have developed some complex adaptive strategies. Recently, ABA and ABA-mimicking ligand, AM1, have been reported to play a crucial role in plants defense against cold stress (Verslues and Zhu, 2005; Cheng et al., 2016; Kim et al., 2016). However, the mechanisms of ABA response to cold stress in Bermudagrass are still largely unknown. In the current study, different physiological mechanisms of exogenous ABA were investigated between cold-sensitive and cold-resistance Bermudagrass genotypes response to cold stress, including cell damage, the process of photosystem II and the alterations of δ13C value.

Cell membrane stability is considered to be a reliable indicator of biotic and abiotic stress- induced cellular damage (Premachandra et al., 1992). The results of this study showed that the leaf EL increased significantly under cold stress, and this increasing rate was higher in the S than the R genotype. Exogenous application of ABA plays an effective role in lowering EL level in the both Bermudagrass genotypes. Interestingly, EL decrease was more in S genotype than R genotype, while the level in the R genotype was still lower than S genotype (**Figure 2B**). This observation resonates with the results of Kumar et al. (2008), who reported that ABA-treated plants had lower EL than those of water-treated plants. The MDA content can be used to evaluate the extent of lipid peroxidation, which further reflects the extent of oxidation injure to the cellular damage (Södergren et al., 2001), and lipid peroxidation was induced by cold stress in many plants (Zhang et al., 2006). Similar to the EL, cold stress significantly increased the MDA content in leaves, which was dramatically decreased by exogenous ABA application in the both genotypes (**Figure 2A**). This observation suggests that cold stress leads to lipid peroxidation, and exogenous ABA played a positive role in maintaining cell membrane stability and normal function, which is consistent with Guschina et al. (2002), who reported that ABA may decrease membrane damage in mosses by diminishing the lipid change.

Reactive oxygen species induced oxidative damage and cell membranes injury (Fridovich, 1978; Sharma et al., 2012). Under cold stress, there is a cellular increase in the superoxide and hydroxyl free radicals which increase membrane lipid peroxidation, leading to cellular damage and destruction (Mittler, 2002). H_2_O_2_ is one of the ROS, and previous research indicated an increase in the amount of leaf H_2_O_2_ when plants were subjected to low temperature (Wise and Naylor, 1987; Hodgson and Raison, 1991; Prasad et al., 1994). In the present study, cold stress dramatically induced the H_2_O_2_ accumulation compared to the control under normal condition, and it was lower in R genotype than S genotype. However, after exogenous application of ABA in the plants of two genotypes, the H_2_O_2_ accumulation significantly decreased (**Figure 3**). This observation suggests that ABA played a crucial role in alleviating oxidation damage during cold stress, which is consistent previous reports from Kumar et al. (2008) and Guo et al. (2012), who reported that exogenous ABA eased oxidative damage of cell membrane by increasing the activity of ROS enzymes under cold stress.

Chlorophyll *a* fluorescence has been widely studied under biotic and abiotic stress (Chen et al., 2013). Cold stress has been reported to decrease photosynthetic electron transport efficiency in plant (Hodgson and Raison, 1989). In this study, alteration of OJIP transient curves suggested that exogenous ABA treatment could enhance cold stress resistance in both genotypes, particularly for the R genotype (**Figure 4**). As an indicator of plant vitality, PI is a sensitive parameter of JIP-test in evaluating plant photochemical activities under stress condition. It combines several parameters that are evaluated through the fluorescence OJIP (Yusuf et al., 2010), and a strong correlation was found between PI and physiological parameters in several studies on stress (Strasser et al., 2007; Zubek et al., 2009). In the study, PI_ABS_ and PI_total_ decreased significantly under cold stress, and they were strongly improved by exogenous ABA in both genotypes, suggesting that exogenous ABA plays a shielded role in cold resistance of two Bermudagrass. Furthermore, cold stress significantly influenced the photosynthetic efficiency, such as decreased the values of φP0, φE0 and 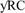, and they were partly recovered by ABA in Bermudagrass (**Table 2**). These data implied that ABA enhances the quantum yield of the electron transport flux on the sides of donor and acceptor of PS II in both Bermudagrass, which is favorable for maintenance high-level photosynthetic property in response to cold stress.

When the rate of energy absorption by chlorophyll exceeds utilization capacity of the organism, the tapped energy is used in photosynthesis or dissipated as heat and ROS are formed (Huner et al., 1998; Pospíšil, 2012). When plants were exposed to cold stress, high PS II excitation pressure caused photosynthetic acclimatization due to the decline in PS II antenna size, which in return reduces photosynthetic efficiency (Huner et al., 1998). Our study showed that exogenous ABA application reduced the values of ABS/RC, RE0/RC and DI0/RC for two genotypes, indicating ABA restrained the cold-triggered energy absorption, trapping and dissipation hence reduced the excessive energy production. Interesting, ET0/RC significantly increased in the R genotype under low temperature, while the values decreased in the S genotype (**Table 2**). This suggested that exogenous ABA reduced the energy flow for electron transport in the R genotype to weaken photosynthesis in response to cold stress. This could be associated with the inherent photosynthesis intensity of the R genotype, which may confer stronger resistance to low temperature.

The inhibition of photosynthesis under cold stress may be attributable to damage to enzyme, thus affecting carbon isotopic fractionation of plants with the photosynthesis (Berry and Bjorkman, 1980; Farquhar, 1983). Bermudagrass is a C_4_ perennial species, and previous studies have showed that C_4_ plants have a δ13C value of -9‰ to -14‰ (Bender, 1971; Smith and Epstein, 1971). However, in this study, the values of δ13C are about -19‰ in normal condition for both genotypes (**Figure 5**), which probably due to growth of the plants in a glasshouse for a long time, where CO_2_ concentration was higher than the that of the air. The finding corresponds to Smith and Boutton (1981), who reported that δ13C values decreased as CO_2_ concentration increased. Conditions that induced stomatal closure, confined the CO_2_ supplement to carboxylation sites, which increased the δ13C values of plants (Farquhar and Richards, 1984; Rivelli et al., 2002; Araus et al., 2003; Yousfi et al., 2012). Low temperature induced stomatal closure, and ABA combined with receptors on the outside of the stomatal guard cell membrane, which further induced stomatal clusore (Cornic and Ghashghaie, 1991; Assmann and Shimazaki, 1999). Therefore in theory, exogenous ABA application may weaken the carbon isotopic fractionation of plants. However, in our study, cold stress increased δ13C values in both genotypes, which were alleviated by ABA. Prior research has identified that the activities of several photosynthetic enzymes, such as PEP-carboxylase, RuDP-carboxylase, were found to be higher in the presence of ABA (Sankhla and Huber, 1979), which could reduce δ13C values via enhanced carbon isotopic fractionation. Moreover, Farquhar et al. (1982) identified that the fractionation caused by diffusion was only 4.4‰, however, the fractionation caused by carboxylation reactions peaked to 30‰. Therefore, the effect of diffusion on stoma could almost be ignored compared to the carboxylation reactions. These results illustrated that ABA heightened the carbon isotope fractionation in both Bermudagrass under cold stress, which may be related to the interaction of stomatal closure and enzyme activities of photosynthesis. However, the regulation of carbon isotope fractionation by ABA is extremely complex under cold stress, therefore, the detailed mechanism still needs further investigation.

As an important plant hormone, ABA has been reported to regulate the expression of many genes. In our study, both cold condition and ABA treatment directly triggered expression level of *ABF1*, and the level in the R genotype was higher than that of S genotype, suggesting that *ABF1* plays a positive role of ABA pathway, especially for the R Bermudagrass (**Figure 6A**). This result is consistent with previous reports from Cheng et al. (2016), who reported that ABF1was up-regulated by AM1 pretreatment and cold stress. Thomashow (1999) and Chinnusamy et al. (2007) has reported that *CBF* genes play vital roles in cold stress. As expected, our findings showed that *CBF1* expression was up-regulated in response to cold stress, which was similar to the study of Shi et al. (2014). In addition, the cold-induced up-regulatiod was generally enhanced by ABA treatment, and the R genotype showed higher levels than the S genotype, indicating that exogenous ABA accelerate *CBF1* gene expression, thus promoting *COR* genes transcription and enhancing cold tolerance of Bermudagrass, especially for the R genotype (**Figure 6B**).

Previous studies have suggested that LEA class proteins are highly hydrophilic, which may function to maintain water and protect macromolecules in dehydrated cells (Chakrabortee et al., 2007). In addition, the LEA protein can function as an antioxidant under water stress, and ABA promotes the synthesis of LEA class proteins. In the present study, under cold stress, the *LEA* gene was generally up-regulated, and the cold-induced up-regulation was enhanced by ABA application, suggesting that exogenous ABA enhanced the transcription of *LEA* and increased accumulation of the LEA protein (**Figure 6C**).

## Conclusion

In summary, exogenous ABA alleviated cold caused oxidative damage in both Bermudagrass genotypes, and more prominently in the R genotype. This may be related to maintenance of cell membrane stability, improvement of the process of photosystem II, increase of carbon isotopic fractionation.

## Author Contributions

LC and JF conceived and designed the experiments. XH, LC, ZH, and AL analyzed the data. XH performed the experiments and wrote the manuscript. LC, HS, ZH, and EA helped to revise the manuscript. All authors read and approved the final manuscript.

## Conflict of Interest Statement

The authors declare that the research was conducted in the absence of any commercial or financial relationships that could be construed as a potential conflict of interest.
